# Unpacking GenAI-enabled deep learning engagement: role perceptions, human–GenAI synergy strategies, and underlying mechanisms

**DOI:** 10.3389/fpsyg.2026.1881713

**Published:** 2026-06-18

**Authors:** Qian Gao, Hui Zhou, Hang Lu

**Affiliations:** 1 School of Urban Management, Beijing Open University, Beijing, China; 2 Academic Affairs Office, Beijing Open University, Beijing, China; 3 School of Education, Zhejiang Normal University, Jinhua, Zhejiang, China

**Keywords:** deep learning engagement, GenAI role perception, human–GenAI synergy, latent profile analysis, metacognition

## Abstract

**Background:**

Generative AI is rapidly transforming how students learn, yet its impact on deep learning varies significantly among individuals. This study investigates the mechanisms linking students’ role perception of GenAI, their human–GenAI synergy strategies, and deep learning engagement.

**Methods:**

A mixed-methods design was employed to analyze data from 181 undergraduate students in a blended course, integrating qualitative coding of reflection reports with quantitative path analysis and latent profile analysis.

**Results:**

Human–GenAI synergy strategies fully mediate the relationship between role perception and deep learning engagement. A critical cognitive threshold was identified where high-order metacognitive engagement emerges primarily when students perceive GenAI as a thinking collaborator rather than a basic efficiency tool. Latent profile analysis revealed six heterogeneous learner profiles, exposing a skill-cognition decoupling phenomenon where complex tool manipulation does not necessarily translate to deep cognitive engagement. Furthermore, an assessment paradox was uncovered, where highly engaged exploratory students often received lower traditional academic scores compared to pragmatic, tool-dependent users.

**Discussion:**

It is concluded that fostering genuine deep learning engagement in the digital era requires shifting pedagogical focus from technical skill training to reshaping students' GenAI role perceptions and updating assessment paradigms to recognize and reward process-oriented human-GenAI metacognitive synergy.

## 1 Introduction

Amidst the wave of digital transformation, guiding students to transition from fragmented surface learning to deep learning—characterized by higher-order thinking and knowledge transfer—remains a core challenge in higher education. Deep learning is employed herein in its pedagogical and cognitive sense—referring to students’ deep approaches to learning and high-order conceptual processing. Generative Artificial Intelligence (GenAI) deeply integrates technology, teaching, and learning, actively reshaping the new ecology of education (O’Dea and O’Dea, 2023) and providing an unprecedented opportunity to address this challenge. As a cognitive tool equipped with generative and conversational capabilities, GenAI plays a vital role in strengthening students’ foundational cognitive skills (Dwivedi et al., 2021; Lim et al., 2023). GenAI not only offers personalized learning scaffolds (Srinivasa et al., 2022; Khalil et al., 2024) and assists learners in rapidly retrieving and summarizing relevant information (Cascella et al., 2023), but it can also stimulate critical thinking capacity through Socratic dialogue, particularly in tasks demanding lower-level cognition (Essien et al., 2024). Theoretically, GenAI-enabled learning environments are associated with enhanced students’ learning engagement and may facilitate the qualitative leap from mere knowledge acquisition to meaning-making.

However, technological empowerment does not occur spontaneously. Studies have pointed out that a substantial number of students merely perceive GenAI as an efficiency tool, utilizing it to quickly generate answers or circumvent cognitive load. From a pedagogical perspective, although this tendency toward Instrumentalism can temporarily improve task completion efficiency, it frequently leads to cognitive inertia and hinders the actual occurrence of deep learning (Dai et al., 2023; Elder et al., 2023). Currently, research on the educational application of GenAI predominantly focuses on quantitative investigations of learners’ initial attitudes, technology acceptance, or usage frequency (Salifu et al., 2024; Strzelecki, 2024; Tan et al., 2024), with some studies also preliminarily exploring surface-level strategies for tool usage. Nevertheless, there remains a notable dearth of micro-level mechanistic research exploring how students subjectively define the role of GenAI (i.e., GenAI role perception) in actual learning processes, what human–GenAI synergy strategies they adopt, and how these two factors jointly impact deep learning engagement. Without clarifying this process mechanism, it is difficult to explain why, under the same learning environments and technical support, students’ depth of learning and engagement quality exhibit immense group differences.

In light of this gap, the present study aims to construct an integrated analytical framework to delve into the intrinsic connections among GenAI role perception, human–GenAI synergy strategies, and deep learning engagement, providing empirical evidence for the reconstruction of human–GenAI collaborative teaching models in higher education. This study adopts a mixed-methods design; it not only focuses on the mediating mechanisms between variables (i.e., how cognition affects learning engagement via strategies) but also reveals the heterogeneous characteristics of student cohorts through Latent Profile Analysis (LPA). Specifically, this study addresses the following core research questions:

*RQ1*: What are the hierarchical characteristics of students’ GenAI role perceptions? How does this cognitive positioning determine the evolution of their human–GenAI synergy strategies?

*RQ2*: What mediating role do human–GenAI synergy strategies play between GenAI role perception and deep learning engagement? Do different cognitive evolutionary pathways exist during human–GenAI interaction?

*RQ3*: Based on human–GenAI synergy strategies and learning performance, what are the heterogeneous profiles of the student cohorts? How do these profiles reveal the validity tension between genuine deep learning processes and traditional academic evaluations?

## 2 Literature review

### 2.1 Generative AI and deep learning

Deep learning is defined as a bidirectional process of constructing knowledge meaning, aimed at enabling learners to acquire a more profound understanding of knowledge (Zhang and Wu, 2012). Distinguished from surface learning, deep learning is a higher-order cognitive processing method characterized by knowledge integration, critical reflection, metacognitive monitoring, and knowledge transfer (Qi et al., 2026). The concept of transformative learning, introduced earlier by Biggs (1979), emphasizes changes in cognitive structures and the reorganization of knowledge, which is highly consistent with the deep understanding and knowledge transformation processes involved in deep learning. In online learning contexts, deep learning entails constructing connections, conducting critical analysis, and integrating concepts across materials and experiences within technology-mediated activities (Akyol and Garrison, 2011). Its ultimate goal is to assist students in establishing meaning, integrating new concepts into existing knowledge maps, and delving into the internal correlations among various components of learning materials (Pegrum et al., 2015).

Regarding the evaluation of deep learning effectiveness, existing research predominantly diverges into process- and outcome-based perspectives. Process-based evaluations mostly utilize questionnaires to measure learning approaches (Biggs et al., 2001) or employ self-reflective logs to record metacognitive development (Bu et al., 2022). However, relying solely on self-reports is insufficient to comprehensively reflect the complex meaning-making that occurs during the learning process (Aguiar-Castillo et al., 2021). Therefore, a scientific evaluation system must integrate learning outcomes, encompassing problem-solving abilities and task completion performance (Webb, 2007). Accordingly, this study proposes that students’ deep learning levels should be comprehensively evaluated across multiple dimensions by combining their self-reports with actual assignment texts.

The intervention of GenAI provides a technological lever to deepen this process. GenAI can not only generate personalized educational content and real-time feedback tailored to individual needs and dynamic changes (Qadir, 2023), but its high adaptability also allows learners to customize interaction styles, thereby optimizing the learning experience (Salminen et al., 2024). Studies have confirmed that such personalized feedback targeting learners’ comprehension levels can significantly enhance academic achievement (Chen et al., 2020; Koyuturk et al., 2023). However, technological empowerment is not without its costs. If learners lack advanced cognitive skills, they are highly susceptible to over-relying on AI feedback, leading to a tendency toward shallow cognition that ironically hinders the cultivation of deep capabilities (Bahrini et al., 2023; Farrokhnia et al., 2023). A systematic review by Zhai et al. (2024) indicates that such over-reliance negatively impacts students’ decision-making and critical thinking abilities. Furthermore, considering that GenAI may generate information that appears confident but is factually incorrect (Lim et al., 2023), learners’ critical judgment during the synergy process becomes particularly crucial.

### 2.2 GenAI role perception

This study defines GenAI role perception as the learners’ subjective understanding and mental representations of the functional attributes, social relations, and metaphorical meanings that GenAI assumes in the learning process. This essentially reflects the students’ epistemic stance toward GenAI. Distributed cognition theory posits that differences in how subjects define tools directly determine the way cognition is distributed.

From the perspective of technology acceptance, the Technology Acceptance Model (TAM) proposed by Davis (1989) argues that perceived usefulness is a key variable explaining technology adoption, which is directly correlated with an individual’s attitude toward the technology (Davis, 1989). Viewed through this lens, some learners treat the machine purely as a tool and focus on the efficiency of output generation, rendering GenAI an efficiency tool. However, from deeper pedagogical and cognitive perspectives, significant hierarchical differences exist in role perceptions: on the one hand, GenAI is viewed as an assistant that can replace teachers to complete teaching tasks highly efficiently and accurately; on the other hand, advanced learners regard it as a cognitive partner, emphasizing the intellectual gaming and dialectic that exist between human and machine.

This variation in role perception directly impacts the investment of metacognition. As an advanced learning strategy, metacognition is vital for realizing deep learning (Bransford et al., 2000). When students view AI merely as a tool, they may neglect metacognitive monitoring; conversely, when they view it as a partner, they are more likely to activate the regulation of their cognitive processes.

### 2.3 Human**–**GenAI synergy strategies

Human–machine collaborative learning theory originates from synergetics (Haken, 1977), which emphasizes that human learners and AI systems collaboratively accomplish knowledge construction through dynamic interaction. Self-Regulated Learning (SRL) theory further stresses that the occurrence of deep learning depends on learners’ active monitoring and regulation of strategies according to task contexts (Winne and Hadwin, 1998).

In collaborative practice, strategies exhibit a distinct morphological evolution. Dai et al. (2025) proposed four stages: human–machine interaction, human–machine fusion, human–machine co-creation, and human–machine symbiosis. Human–GenAI synergy strategies manifest qualitative differences across these stages. In the lower-order stages of human–GenAI synergy, because GenAI lacks an intrinsic mechanism to deepen learners’ cognitive levels, it merely maintains a question-and-answer relationship unless effectively intervened upon (Li and Wang, 2023). A study by Pallant et al. (2025) found that when students utilize GenAI in a procedural and mechanical manner—accepting surface-level information without supplementing it with their own understanding—their learning outcomes actually decrease, stagnating within their existing capability range.

In higher-order human–GenAI synergy, learners assume key cognitive responsibilities such as problem definition, value judgment, and decision-making trade-offs, while GenAI is responsible for providing diverse information and heuristic follow-up questions (Hao and Gu, 2025). This strategy involves the creation and resolution of cognitive conflicts: by introducing heterogeneous information or divergent perspectives, GenAI pushes learners to actively adjust and reorganize their cognitive structures (Tang and Zhang, 2026). In complex problem-solving, this synergy manifests as GenAI continuously providing feedback and presenting multiple potential pathways, while the learner constantly adjusts their understanding based on changing contextual scenarios, collaboratively achieving decision-making (Chen et al., 2023).

## 3 Materials and methods

### 3.1 Research context and participants

This study was grounded in a Social Psychology course at a university in China, with data collected during the fall semester of 2025. The course spanned 10 weeks. The participants consisted of 181 undergraduate students. All participants signed informed consent forms, and the data were anonymized prior to analysis. This study adopted an explanatory sequential mixed-methods design. The instructional design explicitly integrated GenAI as a cognitive scaffold throughout the entire teaching process, encouraging students to utilize tools such as Doubao, DeepSeek, and ChatGPT to assist in completing three phased assignments. This design provided a highly ecologically valid experimental setting for observing how students define GenAI roles and adopt human–GenAI synergy strategies in an authentic educational ecosystem. By transforming rich qualitative reflection texts into structured quantitative codes, we integrated the depth of process-oriented inquiry with the rigor of statistical modeling, thereby achieving a methodological triangulation to examine the human–GenAI synergy ecosystem.

### 3.2 Data collection procedures

This study constructed a multimodal dataset comprising behavioral data, reflective texts, and academic performance. Data collection followed three stages.

First, formative reflection collection required students to submit a human–GenAI Synergistic Learning Reflection Report, in which they structurally reviewed their specific methods of using AI (e.g., prompting strategies), the iterative interaction process, and their critical thinking regarding the AI-generated content during task resolution.

Second, assignment text collection involved gathering the texts of the three course assignments submitted by the students for subsequent thematic analysis and evaluation of deep learning engagement.

Third, academic performance recording was conducted by exporting students’ mastery of knowledge points (based on the accuracy of objective questions in chapter quizzes) and total course grades from the Learning Management System, with the total grade calculated as a weighted average of the three assignments (weighted at 30, 30, and 40% respectively) to serve as an explicit indicator of academic performance.

### 3.3 Measures and coding frameworks

Based on distributed cognition theory and self-regulated learning theory, and drawing on the hierarchical progression logic of the SOLO taxonomy, this study developed a multidimensional coding framework to transform qualitative texts into quantifiable indicators. To ensure scoring reliability, the coding categories were rigorously developed based on existing literature of educational taxonomies. Prior to formal coding, two independent coders underwent a standardized training session. Two researchers independently pre-coded a random sample of 27.6% (*N* = 50). The calculated Cohen’s Kappa coefficient was 0.83, indicating excellent inter-rater reliability for the coding framework. Crucially, the coders were fully blinded to the students’ academic performance outcomes and final grades to eliminate potential halo effects. Disagreements during the coding process were systematically resolved through consensus-seeking meetings facilitated by a third senior educational psychology researcher. The specific coding system is presented in Table 1.

**Table 1:** Coding framework for GenAI role perception and synergy strategies.

Variable	Code	Definition	Example
GenAI role perception	R-0: Non-use/weak tool	Lacks awareness of AI functions or rarely uses AI in learning	I rarely use AI; I feel it’s more reliable to write by myself.
	R-1: Efficiency tool	Views AI as a shortcut to quickly retrieve information or generate standard answers, emphasizing time-saving	AI is like a faster search engine that helps me find materials.
	R-2: Knowledge tutor	Views AI as a tutor to explain concepts and provide examples, showing psychological dependence	When I do not understand a theory, I ask AI to give me an example to explain it.
	R-3: Thinking collaborator	Views AI as a conversational partner to inspire perspectives and fill blind spots, emphasizing heuristic value	I look at how AI analyzes this problem, which gives me a new entry point.
	R-4: Expert partner	Views AI as an expert consultant to solve complex real-world problems, establishing a symbiotic relationship	I treat AI as my consultant to jointly explore community governance plans.
Human**–**GenAI synergy strategies	S-1: Tool dependent	Directly copies and pastes AI-generated content without substantive processing	I sent the prompt to AI and then submitted its answer directly.
	S-2: Concept auxiliary	Uses AI for single-turn Q&A to verify understanding or seek explanations	I asked AI what a ‘stereotype’ is, and after reading its explanation, I understood.
	S-3: Critical integrative	Conducts fact-checking on AI outputs and dialectically integrates them with personal views	Although AI provided a plan, I found it too general, so I revised its logic based on the textbook.
	S-4: Metacognitive synergistic	Uses AI to monitor thinking processes, engaging in multi-turn follow-ups and logical dialectics	I refuted AI’s viewpoint and asked it to regenerate. Through the debate, I clarified my own thoughts.
	S-5: Tool chaining	Proficiently combines multiple AI tools or functions to form a complex technical workflow	I first use Kimi to read literature, then GPT to outline, and finally polish it myself.

GenAI role perception represents students’ stable epistemic beliefs and mental frames—addressing what GenAI is to the learner. In contrast, human–GenAI synergy strategies describe active, self-regulated behavioral workflows—addressing how the learner interacts with GenAI. While logically sequential, they measure distinct cognitive and behavioral domains. This conceptual and empirical distinctiveness is further demonstrated by our empirical findings (see Section 4.1), which show that a high-level behavioral strategy (e.g., S-5) does not automatically entail an advanced cognitive role perception (such as R-4), thereby ruling out construct redundancy.

During the quantitative transformation process, this study not only typologically classified the human–GenAI synergy strategies (S-1 to S-5) but also scored the Strategy Maturity of each reflective text on a 5-point Likert scale, based on the cognitive complexity of the human–machine interaction and learner agency (1 point represents extremely low interaction depth and complete machine dependency; 5 points represent extremely high cognitive complexity and deep metacognitive synergy). This continuous variable was extracted for subsequent statistical modeling.

Regarding the dependent variable, Deep Learning Engagement, this study operationalized it as a higher-order cognitive and reflective practice. Drawing on Mezirow (1991) transformative learning theory and Hatton and Smith's (1995) reflection levels, researchers comprehensively scored the students’ assignments and reflective texts across the following five dimensions (0–2 points per dimension, maximum total score of 10): Application (accurately applying disciplinary concepts to explain phenomena), Authenticity (elaborating by integrating personal experiences or authentic contexts), Complexity (constructing an analytical framework with multiple factors and perspectives), Transfer (proposing specific, actionable problem-solving strategies), and Metacognition (clearly presenting the process of cognitive transformation and mindset monitoring).

For the five dimensions of deep learning engagement (D1–D5), Intraclass Correlation Coefficients (ICC) ranged from 0.87 to 0.94, with the total score ICC = 0.94 (95% CI [0.89, 0.97]), indicating excellent reliability (Koo and Li, 2016). All discrepancies were resolved through discussion.

### 3.4 Data analysis

The data analysis process of this study followed a progressive logic from a variable-centered to a person-centered approach, divided into three stages.

First, descriptive statistics and difference testing were conducted as part of the variable-centered analysis. To verify whether hierarchical effects exist in GenAI role perceptions, a One-Way Analysis of Variance (ANOVA) was employed to examine the differences in strategy maturity among different role perception groups (R-0 to R-4). Tukey’s HSD test was utilized for *post hoc* multiple comparisons to identify the critical cognitive thresholds associated with strategy upgrades.

Second, mechanism exploration and path analysis were carried out as the second part of the variable-centered analysis. To deconstruct the underlying mechanisms among GenAI role perception, human–GenAI synergy strategies, and deep learning engagement, this study employed Ordinary Least Squares (OLS) regression to construct a path analysis model. Prior to analysis, all continuous variables were standardized using Z-scores. Specific steps included: (1) Mediation mechanism testing, focusing on the significance of the direct effect and estimating the indirect effect interval using a bias-corrected Bootstrap method (5,000 resamples); (2) Validity comparison and scissors-difference testing, utilizing Steiger’s *Z*-test to compare the differences in correlation coefficients between strategy maturity and deep learning engagement, and between strategy maturity and academic performance, thereby statistically verifying the validity tension within the evaluation system.

Third, learner profile identification was performed using a person-centered analysis. To reveal the internal heterogeneity of the student cohorts, Latent Profile Analysis (LPA) was conducted. This study utilized a Gaussian Mixture Model (GMM), specified with a full covariance structure to robustly account for potential violations of the local independence assumption. The input variables included the standardized scores of strategy maturity, deep learning engagement, and academic performance. The optimal number of classes was determined based on indicators such as BIC (Bayesian Information Criterion), AIC (Akaike Information Criterion), and Standardized Entropy (where values closer to 1.0 denote higher classification certainty), alongside average posterior probabilities and theoretical interpretability, while carefully considering the stability and limitations of small-sized profiles, thereby constructing accurate learner profiles.

## 4 Results

This study aims to uncover the evolutionary pathways of human–GenAI synergy strategies and their impact on deep learning engagement. This section first adopts a variable-centered approach to elucidate the underlying mechanisms by which learners’ GenAI role perceptions and human–GenAI synergy strategies influence deep learning engagement. Subsequently, it adopts a person-centered approach based on Latent Profile Analysis to reveal the heterogeneous profiles of the student cohorts during the human–GenAI synergy process.

### 4.1 Relationship between role perception and synergy strategies

This study initially explored the characteristics of students’ GenAI role perceptions and their relationship with human–GenAI synergy strategies. The results of the One-Way Analysis of Variance (ANOVA) (see Table 2) showed that role perception had a substantial main effect on strategy maturity (*F*(4, 176) = 56.73, *p* < 0.001, *η*^2^ = 0.56). Tukey’s HSD *post hoc* test revealed that when cognition transitioned from R-2 (Knowledge Tutor) to R-3 (Thinking Collaborator), strategy maturity exhibited the greatest increase (an increase of 1.35 standard deviations, *p* < 0.001). Furthermore, there was no significant difference between R-3 and R-4 (*p* = 0.21), indicating that R-3 acts as a critical cognitive threshold associated with strategy upgrading. Interestingly, the R-3 cohort scored slightly higher in strategy maturity and deep learning engagement than the R-4 cohort, suggesting that viewing AI as a thinking partner rather than an expert consultant is more effective in facilitating advanced collaborative engagement.

**Table 2:** Differences in strategy maturity by GenAI role perception.

GenAI role perception	*n*	Strategy maturity (*M* ± SD)	Deep learning engagement (*M*)	Academic performance (*M*)
R-0: Non-use/weak tool	11	1.00 ± 0.00	6.09	54.17
R-1: Efficiency tool	4	2.00 ± 0.00	6.25	76.78
R-2: Knowledge tutor	113	2.97 ± 0.65	8.20	85.84
R-3: Thinking collaborator	25	4.32 ± 0.48	9.96	86.04
R-4: Expert partner	28	4.11 ± 0.50	9.82	90.02

To further reveal the typological correspondence between role perception and human–GenAI synergy strategies, Table 3 presents their cross-tabulation matrix. The strategies and role perceptions exhibited a clear hierarchical correspondence. The lowest-order collaboration strategy (S-1) absorbed all users with non-existent or weak role perceptions (R-0, 40.7%; R-1, 14.8%), while also containing a portion of R-2 users (44.4%). As strategy complexity increased to S-2, users became highly concentrated in the R-2 (Knowledge Tutor) cohort, accounting for 94.7%. As the key transitional mode, S-3 (Critical Integrative) users were distributed across R-2 (31.3%), R-3 (31.3%), and R-4 (37.5%). The higher-order S-4 (Metacognitive Synergistic) was entirely composed of cohorts with advanced role perceptions: R-3 (76.0%) and R-4 (24.0%).

**Table 3:** Cross-tabulation of human–GenAI synergy strategies and role perception (%).

Human–GenAI synergy strategy	R-0	R-1	R-2	R-3	R-4
S-1: Tool dependent	40.7	14.8	44.5	0	0
S-2: Concept auxiliary	0	0	94.7	1.3	4.0
S-3: Critical integrative	0	0	31.2	31.3	37.5
S-4: Metacognitive synergistic	0	0	0	76.0	24.0
S-5: Tool chaining	0	0	65.8	0	34.2

It is worth noting that among students adopting the S-5 (Tool Chaining) strategy, a distinct cognitive split emerged: although 34.2% of the students reached an R-4 (Expert Partner) role perception, a substantial majority—65.8%—remained at the lower R-2 (Knowledge Tutor) level. This objective data provides compelling evidence for skill-cognition decoupling, indicating that mastering complex, multi-tool combination skills does not necessarily drive an upgrade in individuals’ GenAI role perceptions. Many students operate sophisticated workflows while still fundamentally viewing the AI merely as an advanced tutor.

### 4.2 Mediation effect test of human–GenAI synergy strategies

To examine the mediating role of human–GenAI synergy strategies between role perception and deep learning engagement, this study constructed a path analysis model using standardized regression coefficients and tested the mediation effect based on the Bootstrap method.

The analysis results (see Table 4) showed that GenAI role perception positively predicts human–GenAI synergy strategies (*β* = 0.764, *p* < 0.001), independently explaining 58.4% of the strategy variance (Model *R*^2^ = 0.584). When both role perception and synergy strategies were included to predict deep learning engagement, synergy strategies maintained strong predictive power (*β* = 0.639, *p* < 0.001), whereas the direct effect (*c*′) of role perception plummeted from a total effect of 0.570 to 0.082, and was no longer statistically significant (*p* = 0.321). A Bootstrap test based on 5,000 resamples further confirmed that the indirect effect value was 0.492 (95% CI [0.367, 0.631], excluding 0), with the mediation effect accounting for 86.3% of the total effect. These statistical results conform to a full mediation model, suggesting that higher-order role perceptions appear to operate through their translation into high-level synergy strategies to enhance deep learning engagement.

**Table 4:** Path analysis and mediation effect test.

Hypothesized path	*β*	SE	*t*	*p*	Model *R*^2^
Model 1: Predicting synergy strategy (*M*)					0.584
Path a: Role perception → Synergy strategy	0.764	0.048	15.86	<0.001	
Model 2: Predicting deep learning engagement (*Y*)					0.495
Path c′: Role perception (direct effect)	0.082	0.083	0.99	0.321	
Path b: Synergy strategy	0.639	0.083	7.73	<0.001	
Model 3: Total effect test					0.325
Path c: Role perception → deep learning engagement	0.570	0.061	9.29	<0.001	
Model 4: Validity comparison (predicting performance)					0.081
Synergy strategy → academic performance	0.285	0.072	3.98	<0.001	

*N* = 181. All coefficients are standardized regression coefficients (*β*) calculated based on Z-score standardized data. The significance test for the mediation (indirect) effect utilized the bias-corrected Bootstrap method (5,000 resamples).

Furthermore, a comparison of the models’ explanatory power revealed that synergy strategies could explain 49.5% of the variance in the deep learning engagement model (*R*^2^ = 0.495), but only 8.1% of the variance in the academic performance model (*R*^2^ = 0.081). By comparing the differences in correlation coefficients using Steiger’s *Z* test, the results showed that the correlation between strategy maturity and deep learning engagement (*r* = 0.701) was significantly stronger than its correlation with academic performance (*r* = 0.285), with the difference exhibiting extremely high statistical significance (*Z* = 5.989, *p* < 0.001). This significant discrepancy at the data level objectively reflects that the higher-order process literacies exhibited by students in human–GenAI synergy do not translate proportionately into explicit academic performance under traditional evaluation systems.

### 4.3 Differential characteristics of synergy strategies across dimensions of deep learning engagement

To explore the impact of different human–GenAI synergy strategies on the sub-dimensions of deep learning engagement, a One-Way ANOVA was conducted on the scores of the five synergy strategy patterns (S-1 to S-5) across the five sub-dimensions. The results demonstrated that different strategy patterns exhibited highly significant differences across all five dimensions (*p* < 0.001). Post-hoc multiple comparisons revealed two distinct trajectories of dimension scores changing with strategy patterns (see Table 5).

**Table 5:** Profiles of deep learning engagement dimensions by strategy pattern.

Human**–**GenAI synergy strategy	D1	D2	D3	D4	D5
S-1: Tool dependent	1.70	1.22	1.37	1.19	1.52
S-2: Concept auxiliary	1.92	1.55	1.61	1.33	1.80
S-3: Critical integrative	2.00	1.88	2.00	1.88	2.00
S-4: Metacognitive synergistic	2.00	2.00	2.00	1.92	2.00
S-5: Tool chaining	1.87	1.74	1.76	1.66	1.76
ANOVA *F*	8.23^***^	18.56^***^	15.47^***^	12.91^***^	9.34^***^
*η* ^2^	0.16	0.30	0.26	0.23	0.18

^***^p < 0.001.

The first trajectory manifested among the Tool Dependent (S-1), Concept Auxiliary (S-2), and Tool Chaining (S-5) cohorts. In this sequence, D2 (Authenticity), D3 (Complexity), and D4 (Transfer) all showed an upward trend as strategy complexity increased. The S-5 cohort scored higher on these task-output dimensions than S-1 and S-2, demonstrating exceptionally strong task execution capabilities. However, D1 (Application) and D5 (Metacognition) dimensions presented non-linear fluctuation characteristics. This indicates a double lag in the S-5 cohort regarding precise mastery of fundamental theories (D1) and internal thought monitoring (D5).

The second trajectory manifested between the Critical Integrative (S-3) and Metacognitive Synergistic (S-4) cohorts. Both groups achieved full marks (*M* = 2.00) in the D5 (Metacognition) dimension, significantly higher than other groups. Concurrently, the S-4 cohort achieved the highest score in the D4 (Transfer) dimension (*M* = 1.92), significantly outperforming the S-5 cohort. The data indicates that the S-4 cohort achieved comprehensive high scores across all five dimensions.

### 4.4 Heterogeneous learner profiles based on LPA

To comprehensively capture the complex interactions among students’ human–GenAI synergy strategies, deep learning engagement, and academic performance from a person-centered approach, this study employed Latent Profile Analysis to identify learner profiles. As shown in Table 6, model fit comparison demonstrated that the 6-profile model emerged as the optimal classification. The 6-profile solution yielded the minimum BIC (1324.6) and AIC (1262.8). Furthermore, its Standardized Entropy reached 0.98 (where values closer to 1.0 represent higher classification quality), indicating very clean profile separation, with average posterior probabilities above 0.95, making it the optimal classification solution.

**Table 6:** Fit Indices for latent profile analysis models.

Number of profiles (*k*)	Log-likelihood	AIC	BIC	Standardized entropy
2	−712.3	1,440.6	1,463.2	1.00
3	−683.7	1,391.4	1,423.8	0.94
4	−654.2	1,340.4	1,382.6	0.96
5	−630.5	1,301.0	1,353.0	0.95
6	−608.4	1,262.8	1,324.6	0.98

Standardized entropy ranges from 0 to 1; higher values indicate better classification certainty.

Based on the feature means of each profile (see Table 7), six typical learner profiles were identified.

**Table 7:** Core characteristics and composition of learner profiles.

Learner profile	Percentage	Strategy maturity (*M*)	Deep learning engagement (*M*)	Academic performance (*M*)	Average posterior prob.
C0: Pragmatic tool users	16.0%	2.00	6.93	86.08	0.998
C1: Holistic deep learners	36.5%	4.02	10.00	93.61	0.960
C2: Efficient strategists	3.3%	4.17	8.67	95.10	1.000
C3: Unrecognized explorers	5.5%	4.20	9.90	51.03	1.000
C4: Average mainstream	32.6%	3.00	7.86	83.44	0.991
C5: Disengaged learners	6.1%	1.00	6.09	54.17	1.000

First, the four mainstream cohorts, comprising the majority of the sample, exhibited significant linear development characteristics. C1 (Holistic Deep Learners, 36.5%) represented an ideal learning state, ranking high in strategy maturity (4.02), deep learning engagement (10.00), and academic performance (93.61), achieving comprehensive development empowered by technology. Conversely, C5 (Disengaged Learners, 6.1%) ranked lowest across all indicators, facing risks of academic failure. Falling in between, C4 (Average Mainstream, 32.6%) showed moderate levels across strategies, engagement, and performance, representing typical, step-by-step students. Notably, C0 (Pragmatic Tool Users, 16.0%), despite exhibiting low strategy maturity (*M* = 2.00) and deep learning engagement (*M* = 6.93)—primarily demonstrating tool dependency—maintained good academic performance (M = 86.08). The existence of this cohort reaffirms the aforementioned “low strategy, high performance” phenomenon, indicating that simple instrumental usage is sufficient to meet current assessment standards.

Second, the LPA analysis revealed two atypical cohorts. The first is C2 (Efficient Strategists, 3.3%). Although small in number, this cohort achieved the highest academic performance (*M* = 95.10) relying on extremely high strategy levels (4.17), despite their deep learning engagement not reaching its peak (*M* = 8.67). This suggests an efficient task-oriented pathway, where students might be proficient at leveraging GenAI to generate high-quality assignments that meet grading rubrics without investing in extreme metacognitive reflection. The second is C3 (Unrecognized Explorers, 5.5%). This cohort possessed excellent human–GenAI synergy strategies (*M* = 4.20) and deep learning engagement (*M* = 9.90)—matching the C1 holistic cohort in cognitive investment—yet their academic performance was at the failing borderline (*M* = 51.03). This Competence-Performance Paradox hints at the limitations of current evaluation systems. These students might have deviated from standard answers due to over-exploring complex GenAI-generated content, or they may have been penalized with low scores due to blurred boundaries of assignment originality. We acknowledge that these two profiles represent small percentages of the sample, which requires caution regarding profile stability and replicability; hence, these are interpreted as exploratory subgroups.

## 5 Discussion

This study investigated the underlying mechanisms among GenAI role perception, human–GenAI synergy strategies, and deep learning engagement, while also revealing the heterogeneity of student cohorts through Latent Profile Analysis.

### 5.1 The decisive impact of role perception on human–GenAI synergy strategies

Students’ GenAI role perceptions exhibit significant hierarchical characteristics and appear strongly associated with the evolution of their human–GenAI synergy strategies. When students perceive GenAI merely as an efficiency tool or an auxiliary aid, their cognitive offloading is confined to basic memory retrieval and mechanical processing. Conversely, only when they reframe it as a thinking collaborator capable of dialectical dialogue are they more likely to cultivate higher-order critical integration and metacognitive strategies (Javaid et al., 2023), enabling critical evaluation, structured argumentation, and reflective thinking (Lee et al., 2024). A notable variance in strategy maturity is observed alongside the shift from a knowledge tutor to a thinking collaborator (Wang et al., 2024). This reflects the transition from instrumental rationality to a cognitive symbiosis.

Therefore, universities and educators should shift their pedagogical focus from mere technical training to epistemological reshaping, promoting the use of AI as a cognitive partner for stimulating research and innovation, rather than solely as an auxiliary tool for enhancing output efficiency. It is crucial to guide students to critically explore the boundaries of the human–machine relationship, helping them experience the cognitive transformation from depending on AI for answers to co-constructing problems with AI.

### 5.2 The full mediation mechanism of synergy strategies and metacognitive intervention

Human**–**GenAI synergy strategies play a full mediating role between role perception and deep learning engagement, and a phenomenon of operational complexification coupled with shallow thinking exists in human–GenAI interaction. Students who have mastered intricate multi-tool combination strategies, although excelling in task output, exhibit a significant regression in theoretical application and metacognitive dimensions. This decoupling reflects a process of cognitive offloading (Grinschgl et al., 2021; Risko and Gilbert, 2016), where students delegate critical mental steps to multiple LLMs to minimize internal cognitive effort. Rather than engaging in deep semantic evaluation, these students succumb to a form of automation complacency (Parasuraman and Manzey, 2010), assuming output accuracy simply because their pipeline is sophisticated. This ultimately leads to shallow processing of AI-generated content at a surface level. In such a technology-dependent context, students substitute internal thought monitoring with mechanical workflow orchestration (Johnson et al., 2022). In contrast, students exhibiting higher metacognitive synergy are able to proactively generate cognitive conflicts and scrutinize the logical underpinnings of AI, achieving higher academic outcomes and engaging in deep learning (Broadbent and Fuller-Tyszkiewicz, 2018). It is evident that metacognitive monitoring is closely associated with sustaining high-quality learning engagement (Qi et al., 2026).

Thus, in pedagogical interventions, teachers must position metacognitive monitoring as the core of human–GenAI synergy, encouraging students to record and submit iterative interaction logs that question, refute, and refine AI outputs. By strengthening formative reflection, their critical thinking and metacognitive abilities can be enhanced. Concurrently, teachers can provide scaffolding prompts during students’ interactions with AI tools to elevate their digital learning capabilities (Thomann and Deutscher, 2025).

### 5.3 Heterogeneous learner profiles and the reconstruction of assessment paradigms

Student cohorts exhibit significant heterogeneity in their human–GenAI synergy strategies, revealing a phenomenon of assessment distortion in traditional academic evaluation systems (Chaudhry et al., 2023). The study found a significant misalignment between deep learning engagement and traditional academic performance. Many Pragmatic Tool Users achieved high scores with low-level synergy strategies, while Unrecognized Explorers with high metacognitive engagement were undervalued for deviating from pre-set standard answers. This suggests that traditional outcome-oriented assessment may undergo a validity shift, potentially failing to effectively capture the authentic learning process(Overono and Ditta, 2023). While we must be cautious not to overstate this as a systemic failure of all traditional assessments, our data suggest a structural misalignment. This mismatch can be explained by several alternative factors. First, traditional grading rubrics often heavily penalize minor factual inaccuracies or deviations from structured prompts, which are common in highly exploratory, AI-collaborative work. Second, grading variability and rubric limitations may fail to credit the nonlinear and iterative critical thinking processes of C3 students, focusing instead on standardized surface-level outputs. Consequently, traditional assessments risk creating disincentives for high-level exploratory learning.

Educational administrators must therefore reconstruct the existing academic assessment systems (Gorichanaz, 2023; Morjaria et al., 2023). Universities should introduce multimodal assessment mechanisms that comprehensively evaluate students’ multifaceted competencies, including their human-AI synergistic capabilities (Wang and Zhang, 2026). Furthermore, for innovative tasks, flexible rubrics that embrace heterogeneous thinking should be established, relaxing rigid adherence to standardized answers, thereby genuinely incentivizing higher-order human–GenAI synergy at an institutional level.

### 5.4 Limitations and future directions

Although this study employed mixed methods to deeply investigate the complex mechanisms among GenAI role perception, human–GenAI synergy strategies, and deep learning engagement, it primarily relied on the coding of students’ self-reflection reports and assignment texts. Self-report data may be subject to social desirability bias. Future research could incorporate educational data mining techniques to capture objective log files of student interactions with AI tools, triangulating findings with multimodal data to enhance objectivity. Moreover, this study was situated within a single social psychology course at one university under a specific Chinese cultural context, with a relatively limited sample size. Therefore, generalizing these findings to higher education broadly should be done with caution, as institutional cultures and student demographics vary significantly.

Furthermore, while our six-profile latent solution successfully passed the local independence assumption, we must acknowledge the limitations associated with small-sized subgroups. Although these exploratory, non-typical profiles are theoretically meaningful, their small sizes limit statistical power and profile stability; thus, we caution readers against overgeneralizing these latent cohorts and encourage future replications with larger, more diverse datasets.

Additionally, the cross-sectional, observational nature of our research design precludes any strict causal inferences among GenAI role perception, synergy strategies, and learning engagement. Future research should employ longitudinal tracking designs to capture the dynamic developmental trajectories of students as they evolve from novice to expert users.

## 6 Conclusion

This study constructed and validated an integrative mechanism model that delineates the pathway from GenAI role perception through human–GenAI synergy strategies to deep learning engagement, deeply deconstructing the mechanisms by which GenAI facilitates deep learning engagement in higher education. The research confirms that students’ GenAI role perceptions are a preeminent core variable predicting the efficacy of human–GenAI synergy. Only by transcending the limitations of instrumental rationality and establishing technology as a thinking collaborator is deep metacognitive engagement more likely to be fostered.

This study innovatively identifies the misalignment phenomenon of operational complexification and shallow thinking in human–machine interaction, as well as the limitations of traditional educational assessment. Complex AI tool operations do not equate to the development of higher-order thinking. Moreover, traditional standardized assessments not only fail to effectively evaluate learners’ authentic cognitive processes and performance but may also undervalue innovative learners who dare to engage in deep exploration at the human–machine boundary (Kang, 2023). In the future, to promote students’ deep learning engagement in the digital era, it is imperative for higher education to shift its focus from skill training to epistemological reshaping and to establish a new assessment paradigm centered on incentivizing authentic human–machine metacognitive engagement, thereby truly facilitating the critical thinking and collaborative abilities adapted for the digital intelligence age (Chan, 2023).

## Data Availability

The original contributions presented in the study are included in the article/supplementary material, further inquiries can be directed to the corresponding author.
